# Supplementary material for: Evolution of the Gram-Negative Antibiotic Resistance Spiral over Time: A Time-Series Analysis
Source: Antibiotics (Basel). 2021 Jun 17;10(6):734. doi: 10.3390/antibiotics10060734 (PMC8234935; doi:10.3390/antibiotics10060734)
Supplement: Supplementary file 1 [file antibiotics-10-00734-s001.zip › antibiotics-1243688-supplementary.pdf]

Table S1. Changes in relationships found between drug use and cumulated drug resistance of all Gram-negative bacteria over time in vector autoregressive (VAR) models. These models also inclulde use of other drug groups (fluoroquinolones, aminoglycosides as well as cephalosporins and carbapenems where applicable.)

impulse	response	lags and highest magnitude of significant response (CI) in the response horizon									
		2015		2016		2017		2018		2019	
		forward	reciprocal[1]	forward	reciprocal[1]	forward	reciprocal[1]	forward	reciprocal[1]	forward	reciprocal[1]
cephalo-sporin use	cephalo-sporin R	1-12<br>0.352<br>(0.076 – 0.669)	NSR<br>1.967<br>(-0.621 – 3.041)	1-12<br>0.580<br>(0.165 – 0.823)	5-12<br>2.402<br>(0.398 – 3.064)	4-12<br>0.529<br>(0.132 – 0.814)	3-12<br>2.930<br>(1.161 – 3.673)	3-12<br>0.739<br>(0.183 – 0.976)	3-12<br>2.730<br>(1.333 – 3.594)	3-12<br>0.743<br>(0.230 – 0.958)	3-12<br>2.805<br>(0.864 – 3.852)
cephalo-sporin R	carba-penem use	1-12<br>0.684<br>(0.153 – 0.892)	NSR<br>0.027<br>(-0.093 – 0.142)	2-12<br>0.633<br>(0.171 – 0.911)	NSR<br>0.000<br>(-0.128 – 0.115)	1-12<br>0.615<br>(0.105 – 0.920)	NSR<br>-0.065<br>(-0.288 – 0.152)	2-12<br>0.513<br>(0.044 – 0.718)	NSR<br>-0.042<br>(-0.297 – 0.184)	3-12<br>0.434<br>(0.053 – 0.654)	NSR<br>0.009<br>(-0.210 – 0.235)
carba-penem use	carba-penem R	1-2;<br>0.110<br>(0.040 – 0.157)	NSR<br>-0.042<br>(-0.362 – 0.293)	1<br>0.076<br>(0.015 – 0.129)	NSR<br>-0.099<br>(-0.465 – 0.314)	1, 3-12;<br>0.342<br>(0.049 – 0.548)	NSR<br>-0.050<br>(-0.361 – 0.236)	1, 3-12;<br>0.381<br>(0.058 – 0.592)	NSR<br>-0.048<br>(-0.284 – 0.229)	4-12<br>0.352<br>(0.037 – 0.519)	NSR<br>0.014<br>(-0.299 – 0.308)
carba-penem R	colistin use	1-12<br>0.284<br>(0.091 – 0.429)	NSR<br>-0.229<br>(-0.438 – 0.089)	1-12<br>0.250<br>(0.069 – 0.457)	2-12<br>-0.288[2]<br>(-0.472 – -0.028)	1-6<br>0.105<br>(0.046 – 0.144)	3-12;<br>-0.270[2]<br>(-0.425 – -0.023)	1-7<br>0.150<br>(0.069 – 0.183)	6-12<br>-0.279[2]<br>(-0.470 – -0.025)	1-12<br>0.288<br>(0.032 – 0.433)	NSR<br>-0.117<br>(-0.326 – 0.058)

[1] The reciprocal effect means a switched the role of variables, the impulse is the former response and the response is the former impulse, e.g. reciprocal effect of carbapenem resistance on colistin use means the effect of colistin use on carbapenem resistance.

[2] Negative values indicate that the association is inverse, i.e. the impulse leads to significant decrease of the reponse.

CI: 95% confidence intervals; R: resistance; NSR: no significant response over the response horizon

Table S2. Relationships found between drug use and drug resistance of different Gram-negative bacteria by species.

spe-<br>cies	impulse<br>→<br>response	lags and highest magnitude of significant response (CI) in the response horizon									
		2015		2016		2017		2018		2019	
		forward	reciprocal[1]	forward	reciprocal[1]	forward	reciprocal[1]	forward	reciprocal[1]	forward	reciprocal[1]
Escherichia coli	cephalosporin use →cephalo-sporin R	7-12<br>0.141<br>(0.027 – 0.192)	NSR<br>1.419<br>(-0.121 – 2.403)	3-12<br>0.179<br>(0.075 – 0.238)	9-12<br>1.889<br>(0.235 – 2.949)	3-12<br>0.304<br>(0.100 – 0.365)	3-12<br>2.319<br>(0.273 – 3.505)	2-12<br>0.344<br>(0.119 – 0.428)	4-12<br>2.167<br>(0.023 – 3.475)	NSR<br>0.086<br>(-0.032 – 0.171)	NSR<br>0.847<br>(-0.321 – 1.653)
	cephalosporin R →carbapenem use	1-12<br>0.337<br>(0.029 – 0.567)	NSR<br>0.028<br>(-0.016 – 0.057)	1-9<br>0.352<br>(0.053 – 0.585)	NSR<br>0.019<br>(-0.016 – 0.054)	NSR<br>0.087<br>(-0.271 – 0.347)	NSR<br>-0.019<br>(-0.095 – 0.055)	NSR<br>0.169<br>(-0.265 – 0.629)	NSR<br>-0.037<br>(-0.129 – 0.076)	NSR<br>0.224<br>(-0.174 – 0.479)	NSR<br>-0.050<br>(-0.133 – 0.051)
	carbapenem use →carba-penem R	not examined[2]									
	carbapenem R →colistin use	not examined[2]									
Klebsiella spp	cephalosporin use→cephalo-sporin R	1-6<br>0.153<br>(0.015 – 0.240)	NSR<br>1.118<br>(-0.606 – 2.635)	NSR<br>0.318<br>(-0.102 – 0.564)	NSR<br>-0.026<br>(-0.129 – 0.082)	NSR<br>0.141<br>(-0.057 – 0.249)	NSR<br>1.695<br>(-0.167 – 3.103)	NSR<br>0.151<br>(-0.049 – 0.281)	NSR<br>0.738<br>(-0.334 – 1.817)	4-12<br>0.367<br>(0.143 – 0.470)	3-12<br>2.305<br>(0.186 – 3.798)
	cephalosporin R →carbapenem use	NSR<br>0.272<br>(-0.142 – 0.588)	NSR<br>-0.006<br>(-0.153 – 0.118)	NSR<br>0.078<br>(-0.071 – 0.190)	NSR<br>0.163<br>(-0.518 – 0.726)	NSR<br>0.437<br>(-0.019 – 0.662)	NSR<br>-0.025<br>(-0.155 – 0.128)	3<br>0.221<br>(0.019 – 0.397)	NSR<br>-0.012<br>(-0.153 – 0.116)	NSR<br>0.173<br>(-0.205 – 0.566)	NSR<br>-0.035<br>(-0.110 – 0.191)
	carbapenem use→carba-penem R	1-4<br>-0.006[3]<br>(-0.009 – -0.002)	NSR<br>0.057<br>(-0.314 – 0.337)	NSR<br>0.003<br>(-0.006 – 0.014)	NSR<br>-0.170<br>(-0.449 – 0.343)	NSR<br>-0.005<br>(-0.023 – 0.012)	NSR<br>0.094<br>(-0.265 – 0.363)	NSR<br>-0.008<br>(-0.030 – 0.012)	NSR<br>0.099<br>(-0.165 – 0.436)	NSR<br>-0.007<br>(-0.029 – 0.022)	NSR<br>-0.082<br>(-0.351 – 0.409)
	carbapenem R →colistin use	1-12<br>0.172<br>(0.021 – 0.250)	NSR<br>0.004<br>(-0.002 – 0.008)	1-12<br>0.170<br>(0.037 – 0.286)	NSR<br>0.004<br>(-0.004 – 0.010)	1-12<br>0.131<br>(0.010 – 0.214)	NSR<br>0.004<br>(-0.009 – 0.015)	NSR<br>0.061<br>(-0.059 – 0.223)	1-2<br>-0.015[3]<br>(-0.022 – -0.006)	NSR<br>-0.193<br>(-0.361 – 0.034)	1-3<br>-0.021[3]<br>(-0.029 – -0.010)
Pseud<br>omon	cephalosporin use →cephalosporin R	NSR<br>0.102<br>(-0.122 – 0.260)	NSR<br>1.203<br>(-0.249 – 2.131)	NSR<br>-0.043<br>(-0.153 – 0.113)	NSR<br>0.208<br>(-0.674 – 0.831)	NSR<br>0.087<br>(-0.087 – 0.274)	NSR<br>1.241<br>(-0.237 – 2.179)	NSR<br>0.203<br>(-0.066 – 0.391)	3-12<br>2.291<br>(0.377 – 3.259)	NSR<br>-0.205<br>(-0.308 – 0.061)	NSR<br>0.003<br>(-0.133 – 0.127)

	cephalosporin R →carbapenem use	NSR<br>0.251<br>(-0.214 – 0.551)	NSR<br>0.009<br>(-0.090 – 0.113)	6-12<br>0.570<br>(0.115 – 0.690)	NSR<br>0.011<br>(-0.123 – 0.116)	NSR<br>0.386<br>(-0.147 – 0.686)	NSR<br>-0.042<br>(-0.189 – 0.157)	NSR<br>0.230<br>(-0.104 – 0.592)	NSR<br>-0.023<br>(-0.169 – 0.109)	NSR<br>0.109<br>(-0.248 – 0.432)	NSR<br>0.003<br>(-0.133 – 0.127)
	carbapenem use→carba-penem R	NSR<br>0.084<br>(-0.048 – 0.182)	NSR<br>0.332<br>(-0.091 – 0.639)	1<br>0.043<br>(0.012 – 0.074)	NSR<br>0.153<br>(-0.120 – 0.377)	NSR<br>0.047<br>(-0.036 – 0.108)	NSR<br>0.086<br>(-0.128 – 0.282)	NSR<br>0.061<br>(-0.045 – 0.137)	NSR<br>0.132<br>(-0.127 – 0.329)	NSR<br>0.045<br>(-0.028 – 0.114)	NSR<br>0.150<br>(-0.109 – 0.360)
	carbapenem R →colistin use	1-6<br>0.144<br>(0.020 – 0.245)	2-12<br>-0.174[3]<br>(-0.244 – -0.030)	2-12<br>0.207<br>(0.034 – 0.359))	1-12<br>-0.166[3]<br>(-0.239 – -0.048)	NSR<br>0.024<br>(-0.080 – 0.160)	NSR<br>-0.066<br>(-0.150 – 0.049)	NSR<br>0.038<br>(-0.094 – 0.177)	NSR<br>-0.083<br>(-0.142 – 0.026)	NSR<br>0.120<br>(-0.093 – 0.258)	NSR<br>-0.019<br>(-0.077 – 0.053)
Acinetobacter baumannii	cephalosporin use →cephalosporin R	not examined[4]									
	cephalosporin R →carbapenem use	not examined[4]									
	carbapenem use →carbapenem R	1-3<br>0.071<br>(0.025 – 0.107)	NSR<br>-0.192<br>(-0.576 – 0.166)	1-4<br>0.123<br>(0.036 – 0.193)	NSR<br>-0.238<br>(-0.476 – 0.066)	1, 3-12<br>0.252<br>(0.045 – 0.430)	NSR<br>-0.251<br>(-0.486 – 0.113)	1-12<br>0.344<br>(0.097 – 0.507)	NSR<br>-0.207<br>(-0.446 – 0.038)	1, 3-12<br>0.294<br>(0.036 – 0.444)	NSR<br>-0.143<br>(-0.455 – 0.155)
	carbapenem R →colistin use	1-12<br>0.271<br>(0.081 – 0.466)	NSR<br>-0.020<br>(-0.151 – 0.135)	1-2<br>0.096<br>(0.029 – 0.135)	NSR<br>-0.108<br>(-0.223 – 0.074)	1-4<br>0.133<br>(0.056 – 0.179)	NSR<br>-0.113<br>(-0.253 – 0.068)	1-5<br>0.154<br>(0.082 – 0.194)	NSR<br>-0.043<br>(-0.185 – 0.083)	1-12<br>0.317<br>(0.082 – 0.481)	NSR<br>-0.166<br>(-0.320 – 0.060)

[1] The reciprocal effect means that the impulse is the former response and the response is the former impulse, e.g. reciprocal effect of carbapenem resistance on colistin use means the effect of colistin use on carbapenem resistance.

[2] As carbapenem resistance in *E. coli* was extremely rare in the study period, these associations were not tested.

[3] Negative values indicate that the association is inverse, i.e. the impulse leads to significant decrease of the reponse.

[4] As in case of *A. baumannii* cephalosporin susceptibility is not tested and all isolates are interpreted as cephalosporin resistant, these associations were not tested.

CI: 95% confidence intervals R: resistance NSR: no significant response over the response horizon

Table S3. Changes in relationships found over the complete resistance spiral over time. Light shading marks significant relationships, dark shading marks significant reciprocal associations.

impulse	response	lags and highest magnitude of significant response (CI) in the response horizon									
		2015		2016		2017		2018		2019	
		forward	reciprocal[1]	forward	reciprocal[1]	forward	reciprocal[1]	forward	reciprocal[1]	forward	reciprocal[1]
cephalo-sporin use	cephalo-sporin R in *E. coli*	NSR<br>0.013<br>(-0.044 – 0.056)	NSR<br>0.021<br>(-0.619 – 0.521)	NSR<br>0.016<br>(-0.024 – 0.058)	NSR<br>0.223<br>(-0.375 – 0.681)	NSR<br>0.010<br>(-0.052 – 0.075)	NSR<br>0.131<br>(-0.460 – 0.470)	NSR<br>0.002<br>(-0.080 – 0.081)	NSR<br>0.204<br>(-0.269 – 0.557)	NSR<br>0.034<br>(-0.079 – 0.101)	NSR<br>0.827<br>(-0.180 – 1.558)
cephalo-sporin R in *E. coli*	carba-penem use	1-6<br>0.256<br>(0.071 – 0.349)	NSR<br>0.032<br>(-0.021 – 0.069)	1-7<br>0.236<br>(0.055 – 0.307)	NSR<br>0.022<br>(-0.022 – 0.069)	1-3<br>0.171<br>(0.053 – 0.278)	NSR<br>-0.021<br>(-0.072 – 0.060)	NSR<br>0.196<br>(-0.146 – 0.537)	NSR<br>-0.039<br>(-0.113 – 0.082)	NSR<br>0.095<br>(-0.330 – 0.444)	NSR<br>-0.052<br>(-0.123 – 0.026)
carba-penem use	carbap. R in *P. aeruginosa*	NSR<br>0.064<br>(-0.071 – 0.155)	NSR<br>0.118<br>(-0.225 – 0.382)	NSR<br>0.003<br>(-0.096 – 0.083)	NSR<br>0.133<br>(-0.191 – 0.352)	NSR<br>0.029<br>(-0.093 – 0.104)	NSR<br>0.185<br>(-0.170 – 0.425)	NSR<br>0.037<br>(-0.076 – 0.116)	NSR<br>0.159<br>(-0.121 – 0.384)	NSR<br>0.049<br>(-0.042 – 0.120)	NSR<br>0.148<br>(-0.151 – 0.422)
carba-penem use	carbap. R in *A. baumannii*	NSR<br>0.095<br>(-0.089 – 0.197)	NSR<br>-0.297<br>(-0.564 – 0.142)	NSR<br>0.119<br>(-0.029 – 0.229)	2-4<br>-0.195[2]<br>(-0.307 – -0.041)	1-4<br>0.107<br>(0.022 – 0.164)	2-4<br>-0.189[2]<br>(-0.282 – -0.041)	1-12<br>0.292<br>(0.059 – 0.410)	3-4<br>-0.134[2]<br>(-0.292 – -0.017)	1-12<br>0.326<br>(0.046 – 0.424)	NSR<br>-0.045<br>(-0.287 – 0.135)
carbap. R in *P. aeruginosa*	colistin use	NSR<br>0.178<br>(-0.015 – 0.269)	1-12<br>-0.178[2]<br>(-0.226 – -0.024)	3-6<br>0.118<br>(0.011 – 0.217)	1-12<br>-0.183[2]<br>(-0.241 – -0.062)	NSR<br>0.024<br>(-0.121 – 0.142)	2-12<br>-0.126[2]<br>(-0.174 – -0.024)	NSR<br>0.048<br>(-0.084 – 0.204)	NSR<br>-0.096<br>(-0.152 – 0.023)	NSR<br>0.042<br>(-0.116 – 0.136)	NSR<br>-0.056<br>(-0.109 – 0.020)
carbap. R in *A. baumannii*	colistin use	1-6<br>0.112<br>(0.034 – 0.140)	NSR<br>-0.038<br>(-0.162 – 0.101)	1-3<br>0.110<br>(0.035 – 0.140)	NSR<br>-0.087<br>(-0.162 – 0.062)	1-9<br>0.113<br>(0.058 – 0.151)	NSR<br>-0.186<br>(-0.305 – 0.028)	1-10<br>0.193<br>(0.018 – 0.301)	NSR<br>-0.178<br>(-0.317 – 0.056)	1-12<br>0.295<br>(0.096 – 0.360)	NSR<br>0.144<br>(-0.334 – 0.064)

[1] The reciprocal effect means a switched the role of variables, the impulse is the former response and the response is the former impulse, e.g. reciprocal effect of cephalosporin use on cephalosporin resistance means the effect of cephalosporin resistance on cephalosporin use

[2] Negative values indicate that the association is inverse, i.e. the impulse leads to significant decrease of the reponse.

CI: 95% confidence intervals; R: resistance; NSR: no significant response over the response horizon
